# SPP-SegNet and SE-DenseNet201: A Dual-Model Approach for Cervical Cell Segmentation and Classification

**DOI:** 10.3390/cancers17132177

**Published:** 2025-06-27

**Authors:** Betelhem Zewdu Wubineh, Andrzej Rusiecki, Krzysztof Halawa

**Affiliations:** Faculty of Information and Communication Technology, Wroclaw University of Science and Technology, 50-370 Wroclaw, Poland; andrzej.rusiecki@pwr.edu.pl (A.R.); krzysztof.halawa@pwr.edu.pl (K.H.)

**Keywords:** cervical cancer, image segmentation, classification, SegNet, SE-DenseNet201

## Abstract

Background/Objectives: Cervical cancer, the fourth most common malignancy in women worldwide, continues to pose a significant threat to global health. Manual examination of the Pap smear image is time-consuming, labor-intensive, and prone to human error due to the large number of slides and subjective judgment. This study proposes a novel SegNet-based spatial pyramid pooling (SPP-SegNet) deep learning model for segmentation and a Squeeze-and-Excitation-based (SE-DenseNet201) model for classification, aimed at improving the accuracy of cervical cancer detection. Methods: The model incorporates the SPP bottleneck and atrous convolution in the SegNet framework, allowing for the extraction of multiscale spatial features and improving segmentation performance. The segmentation output is used as input for the classification task. The proposed method is evaluated on the Pomeranian and SIPaKMeD datasets. Results: Segmentation results show that SPP-SegNet achieves 98.53% accuracy on the Pomeranian data set, exceeding standard SegNet, 97.86%. It also achieves 94.15% accuracy on the SIPaKMeD dataset, outperforming the standard SegNet, which is 90.95%. For classification, SE-DenseNet201 achieves 93% and 99% accuracy for the Pomeranian and SIPaKMeD binary classification, respectively, using the bounding box input. Conclusions: These results show that SPP-SegNet and SE-DenseNet201 can potentially automate cervical cell segmentation and classification, facilitating the early detection and diagnosis of cervical cancer.

## Simple Summary

Cervical cancer is one of the most common cancers in women, and detecting it early is critical for successful treatment. Currently, doctors examine Pap smear images manually, which takes a lot of time and can lead to mistakes. To help improve this process, we have developed two advanced computer models. One model accurately separates cervical cells from the background in images, while the other classifies whether the cells are normal or abnormal. We tested our method on two well-known image datasets and found that it works better than existing systems in both identifying and classifying cervical cells. These results show that our system could support medical professionals by making cervical cancer screening faster, more accurate, and more consistent. This approach may ultimately help detect cancer earlier and improve patient outcomes.

## 1. Introduction

Cervical cancer is a global health concern, ranking as the fourth most prevalent cancer among women worldwide [1]. In 2018, approximately 570,000 new cases and 311,000 deaths were attributed to cervical cancer, highlighting the urgent need for effective prevention and early detection strategies [2]. The Pap smear test, a widely adopted screening method, involves collecting and microscopically examining cervical cells to detect precancerous or cancerous changes. This test has been instrumental in reducing the incidence and mortality rates of cervical cancer in many developed countries [3,4]. However, manual screening of Pap smear images is labor-intensive, time-consuming, and susceptible to human error due to subjective judgment and the large volume of slides that require analysis [5,6,7].

Automating the Pap smear screening process through image processing and analysis offers a promising solution to overcome these challenges. Image segmentation, a fundamental step in this automation, involves partitioning an image into distinct regions of interest, such as the nucleus and cytoplasm of cervical cells [4]. Accurate segmentation is crucial for subsequent feature extraction and classification tasks, as it facilitates the identification of abnormal cells that may indicate precancerous or cancerous conditions [1,8]. However, segmenting cervical cells in Pap smear images is inherently challenging due to factors such as overlapping cells, variations in stain quality, and the presence of artifacts [4].

Deep learning (DL), a subfield of machine learning, has been widely used in medical image analysis, demonstrating remarkable capabilities in tasks such as image segmentation and classification [2,9]. By learning complex patterns and representations from large datasets, DL models improve the accuracy and efficiency of medical image interpretation [4]. Convolutional neural networks (CNNs), in particular, automatically learn features from images [10], eliminating the need for manual feature engineering, and are especially effective for both segmentation and classification tasks [11,12].

SegNet is a DL architecture specifically designed for image segmentation [13]. It consists of an encoder network that extracts features from the input image and a decoder network that upsamples these features to produce a segmented output [14]. To enhance the performance of SegNet in cervical cell segmentation, we propose a novel approach that integrates a spatial pyramid pooling (SPP) bottleneck and incorporates an atrous convolution (dilation rate) in the encoder. SPP enables the extraction of multiscale features, improving robustness to variations in cell size and shape [15]. The SPP bottleneck is placed between the SegNet encoder and decoder networks to aggregate features at different scales before upsampling. For classification tasks, the squeeze-and-excitation block is incorporated into the DenseNet201 (SE-DenseNet201) model to improve feature recalibration by emphasizing informative features and suppressing less relevant ones.

This study aims to develop a novel framework for cervical cell segmentation and classification using SPP-SegNet and SE-DenseNet201. The contributions are as follows:

- Introduced SPP-SegNet, incorporating spatial pyramidal grouping and atrous convolutions to improve cervical cell segmentation performance.
- Developed SE-DenseNet201, integrating squeeze-and-excitation (SE) blocks to improve feature recalibration and boost classification accuracy.

The remainder of the paper is organized as follows. Section 2 discusses related work, Section 3 describes the materials and methods used in this study, Section 4 presents the results and discusses the findings, and Section 5 provides concluding remarks.

## 2. Related Works

Several research studies have been conducted on automating cervical cancer screening by segmenting and analyzing Pap smear images. Xiong et al. [16] conducted research on automated segmentation methods for the early detection of cervical cancer using DL-based magnetic resonance imaging (MRI). The study focused on segmenting the tumors using a single-shot multi-box detector (SSD), a squeeze-and-excitation block, and a U-Net attention gate (SE-ATT-Unet), achieving a remarkable precision of 92.32%. Chankong et al. [3] proposed a method for automatic cervical cell segmentation and classification using fuzzy C-means (FCMs) clustering and classifiers such as artificial neural networks (ANNs). The study scored a precision value of 85% using the Herlev dataset and 87% using the LCH datasets of nucleus segmentation. Zhu and Du [17] researched the segmentation of overlapping cervical cells based on boundary tracking. The study used the U-Net architecture to segment the image and used the center of each nucleus to identify the boundary point at a different angle. The model scored a precision value of 95.04%.

Furthermore, Beatriz et al. [18] proposed a U-Net and SegNet method to reduce mortality rates due to the implementation of Pap smears and issues related to the hindrance of false positive and negative results in the detection of cervical cancer. In relation to the performance of U-Net and SegNet neural networks for nuclei segmentation, these studies reported significant success, with SegNet achieving an accuracy of 95.52% in real cytological datasets (CRIC Cervix-Seg), demonstrating its ability to handle overlapping structures and artifacts effectively. Furthermore, Song et al. [19] researched overlapping cytoplasm segmentation via constrained multi-shape evolution for cervical cancer screening. The paper proposed an a priori-based shape model to address challenges in overlapping cytoplasm by combining local and global shapes. It yielded a Dice similarity coefficient of 83% in the pap smear image.

SegNet’s encoder-decoder architecture has been adapted for various segmentation tasks. Vianna et al. [20] demonstrated U-Net and SegNet performance on lesion segmentation of breast ultrasound images, achieving a Dice coefficient of 81.1%. Badrinarayanan et al. [21] proposed a SegNet deep convolutional encoder–decoder architecture for pixel-wise semantic image segmentation. The paper highlighted its memory efficiency and competitive performance compared to architectures such as FCN and DeepLab. The model scored an mIoU of 60.1%. Moreover, Brahmbhatt and Rajan [22] worked on skin lesion segmentation using SegNet with binary cross-entropy. The study achieved an intersection over union (IoU) value of 92% with minimal pre- and post-processing using the PH2 dataset. Goker [23] worked on the detection of cervical cancer from images of the uterine cervix using transfer learning. The model achieved an accuracy of 95.09% in the Herlev dataset using DenseNet20.

Although previous studies, such as those of [18], have taken advantage of U-Net and SegNet architectures for cervical cell segmentation, handling overlapping structure challenges, such as limited multiscale feature extraction, have been encountered [17,19]. To address these issues, this study proposes SPP-SegNet and SE-DenseNet201 to achieve superior segmentation and classification accuracy in the Pomeranian and SIPaKMeD datasets compared to the standard SegNet and DenseNet201 methods.

## 3. Materials and Methods

In this section, we describe the dataset, the pre-processing technique, the proposed method, the implementation details, and the performance metrics.

### 3.1. Dataset and Preprocessing Technique

The dataset was obtained from the Pomeranian Medical University in Szczecin, Poland. It comprised 419 images categorized into three classes: high squamous intraepithelial lesions (HSIL), low squamous intraepithelial lesions (LSIL), and normal squamous intraepithelial lesions (NSIL) [24]. The images were originally in BMP format, most of which had a resolution of 1130 × 1130 pixels. Furthermore, we used the publicly available SIPaKMeD dataset, which contains 4049 single-cell images classified into five categories: three representing abnormal cells and two representing normal cells. A summary of the details of the datasets is provided in Table 1.

During pre-processing, the images were resized to 224 × 224 pixels to ensure uniformity and optimize computational efficiency. The intensity values of the pixels were normalized to the range [−1, 1] to improve the stability of the model during training. Additionally, we created masks to segment Pap smear images using OpenCV, where all cells were represented as white and the background as black.

### 3.2. Proposed Method

**Figure 1:**
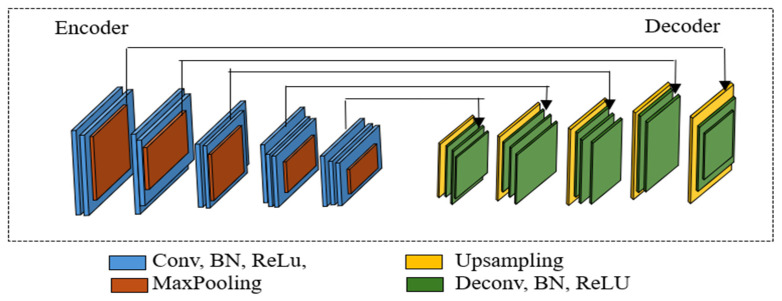
The standard SegNet architecture.

For segmenting whole cells from the background, we employed the SegNet architecture, a well-known pixel-wise semantic segmentation method [25]. Built on the VGG-16 framework, SegNet removes fully connected layers and adopts a symmetric encoder–decoder structure [26]. Its ability to capture both low-level and high-level features, combined with its efficient upsampling strategy, makes it suitable for delineating fine-grained structures in medical images [18]. The standard SegNet model is illustrated in Figure 1.

However, the standard SegNet architecture struggles to capture global or multi-scale context effectively, which is crucial for segmenting objects or cells of different sizes and shapes. Thus, this study proposed a novel approach, SPP-SegNet, which improves the accuracy and efficiency of cervical cell segmentation by combining SegNet with spatial pyramid pooling (SPP) and atrous convolutions to increase the receptive field without increasing the number of parameters. The SPP block was designed to extract multiscale spatial information, improve the receptive field, and preserve fine-grained features for decoding.

In the encoding stage, the model comprised five convolutional blocks, each using atrous convolutions with progressively increasing dilation rates. This configuration expands the receptive field without increasing computational complexity or loss of resolution. The dilation rates were 1 × 1, 2 × 2, 4 × 4, 8 × 8, and 16 × 16 in the last layers, capturing broader contextual information. Batch normalization and ReLU activation were applied after each convolution to ensure stable learning and nonlinear transformations. The MaxPooling layers in each block progressively reduced the spatial resolution, generating feature maps with high-level semantics. The bottleneck integrated an SPP block to capture multi-scale features by pooling the feature maps at different scales.

**Figure 2:**
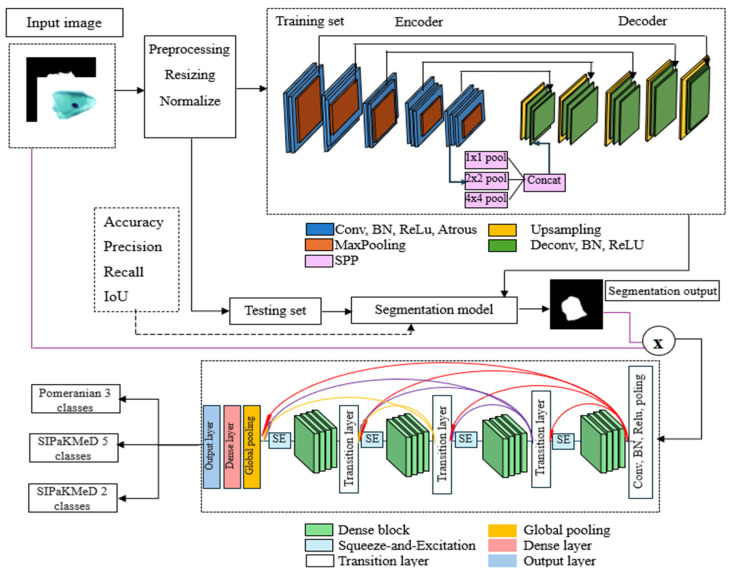
The proposed segmentation and classification method.

The decoder mirrored the encoder structure but included upsampling and transposed convolutions to restore spatial resolution. Upsampling layers are applied in each block to upscale feature maps back to the original input resolution. Transposed convolutions replaced standard convolutions in the decoding stage, reconstructing spatial details. The final layer generated a single-channel output representing the segmented image, using a sigmoid activation function to produce probabilities in the shape of 224 × 224. The proposed model is depicted in Figure 2.

As shown in Figure 2, segmentation and classification tasks were performed using DL architecture. For the classification task, a novel SE-DenseNet201 model was employed, where SE blocks were integrated after every dense block to recalibrate channel-wise feature responses, improving the model’s ability to distinguish subtle variations in cervical cell features and enhance classification accuracy. First, we performed segmentation, followed by classification. For classification, we used two approaches: ROI-based (segmentation output multiplied by the original image) and bounding box (BBox) (determining bounded boxes containing the segmented fragment and cutting out the regions bounded by them from the original images). In the ROI image, the background was black, and the entire cell was extracted in RGB color. In contrast, the BBox approach cropped the RGB color from the original image based on the coordinates derived from the segmentation result.

### 3.3. Training Details and Performance Metrics

This study used Python 3.11 with TensorFlow and Keras on a Windows-based system with an NVIDIA GeForce RTX 3090 GPU. The model was trained using the Adam optimizer with a learning rate of 0.001 for 100 epochs to perform segmentation and classification tasks. A batch size of 18 was selected to balance computational efficiency and model stability, allowing frequent weight updates while optimizing memory usage. We used accuracy, precision, recall, and intersection over union (IoU) as performance metrics to segment all cells from the background. We used accuracy, precision, recall, and F1 score for classification as performance metrics.

The computational cost of the proposed SPP-SegNet method is summarized as follows. It contained approximately 21.9 million parameters, with training conducted over 100 epochs. The average training time per epoch was approximately 5 s, and the inference time per image was around 350 ms. Memory usage remained within acceptable limits for modern GPU-equipped systems. These results suggest that our model is efficient and feasible for deployment in moderately resource-constrained environments.

## 4. Results and Discussions

The following are the experimental setups implemented in this study. We used a novel SPP-SegNet DL architecture to separate entire cells from the background and SE-DenseNet201 to classify cervical cells. To evaluate the proposed model, we used the Pomeranian and SIPaKMeD datasets, which were divided into training, validation, and testing sets. Experiments were conducted using both the standard SegNet and the novel SPP-SegNet models for segmentation. The results of the segmentation for both the standard SegNet and the proposed SPP-SegNet models are presented in Table 2.

As shown in Table 2, the proposed SPP-SegNet model outperforms the standard SegNet across all evaluated datasets. On the Pomeranian dataset, SPP-SegNet achieved 98.53% accuracy, surpassing 97.86% for SegNet, with improved precision (96.41% vs. 91.9%), recall (91.32% vs. 90.31%), and IoU (95.75% vs. 95.55%). These results demonstrate the superior ability of SPP-SegNet to segment and detect relevant features while reducing false positives. Similarly, on the SIPaKMeD dataset, SPP-SegNet achieved 94.15% accuracy, significantly higher than the 90.95% of SegNet. Precision (93.87% vs. 92.40%), recall (94.94% vs. 90.05%), and IoU (95.08% vs. 92.89%) also show considerable improvements, confirming the effectiveness of SPP in enhancing multiscale feature extraction and segmentation performance. On Herlev, SPP-SegNet also demonstrated improved performance, achieving 87.58% accuracy compared to 86.74% for SegNet, along with gains in precision (91.95% vs. 91.05%), recall (91.67% vs. 90.35%), and IoU (91.13% vs. 90.33%). These consistent improvements across three distinct datasets reinforce the robustness and general applicability of the SPP-SegNet model for cervical cell segmentation under varying imaging and morphological conditions.

### Ablation Study

We conducted an ablation study for the classification task to identify the best method for integrating classification with segmentation. In this ablation study, the classification task used the original image (without segmentation), the extracted ROI as input, and the BBox extracted using ResNet50V2, DenseNet121, and DenseNet201, our proposed SE-DenseNet201. The output of the segmentation was used as input for classification, as shown in Table 3.

Table 3 shows the classification performance of three different input methods: the original image, the extracted Region of Interest (ROI), and the BBox method. For the Pomeranian dataset, the BBox method achieved the highest accuracy, outperforming the ROI-based methods by 3% (88% accuracy compared to 85% for ROI). Similarly, for the SIPaKMeD and the Herlev dataset, the BBox method achieved an accuracy of 91% and 88%, respectively, which are comparable to the classification-only result for multiclass and binary classification. These results suggest that BBox extraction provides the most accurate classification results, particularly for the Pomeranian dataset. We then compare these results with the classification results using the proposed SE-DenseNet201 method, as shown in Table 4.

**Figure 3:**
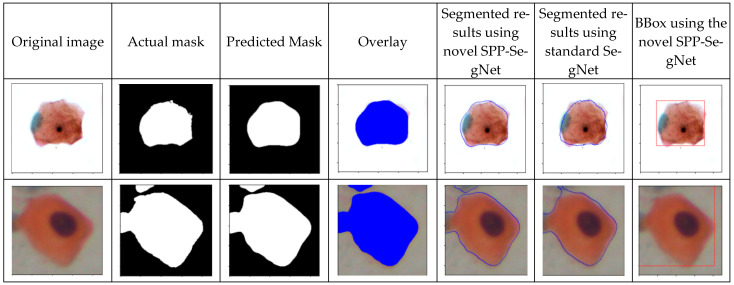
Segmentation results for the Pomeranian and SIPaKMeD dataset.

In Table 4, we can observe that the SE-DenseNet201 method with the BBox consistently outperforms the methods presented in Table 3, demonstrating a notable improvement in classification performance. For the Pomeranian dataset, the SE-DenseNet201 using the BBox method achieved 93% accuracy, significantly improving from results obtained with the standard method (ResNet50V2, DenseNet121, DenseNet201). For the SIPaKMeD multiclass dataset, the SE-DenseNet201 BBox method achieved an accuracy of 96%, outperforming the standard methods in Table 3 by 5%. Similarly, for binary classification, the SE-DenseNet201 BBox model showed an impressive 99% accuracy, precision, recall, and F1 score, surpassing all other methods. In the Herlev dataset, the proposed method achieves an accuracy of 90% using the BBox. These results highlight that the SE-DenseNet201 model, particularly with the BBox input, offers a significant enhancement in classification tasks compared to traditional methods, as shown by the improved metrics in both datasets. This reinforces the effectiveness of the proposed SE-DenseNet201 model, especially when using BBox output from segmentation, which is crucial for improving classification accuracy. In conclusion, the ablation study shows that the SPP-SegNet method, when combined with the proposed SE-DenseNet201, yields the best performance, particularly in terms of classification accuracy. The segmentation output for both the Pomeranian and SIPaKMeD datasets is shown in Figure 3.

**Figure 4:**
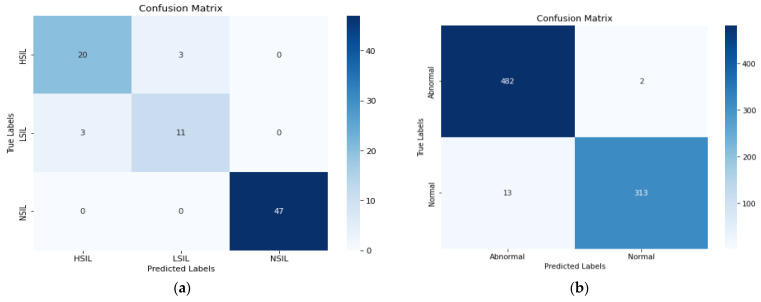
Confusion matrix: (**a**) Pomeranian and (**b**) SIPaKMeD.

As shown in Figure 3, there are two rows for the Pomeranian and SIPaKMeD datasets, respectively. The first column displays the actual image, and the second column shows the ground truth corresponding to each image. In the third column, the predicted outputs are displayed, and in the fourth column, an overlay of the predicted output is shown. The fifth column presents the segmented output using the proposed SPP-SegNet, the sixth column shows the segmented output using the standard SegNet, and the last column shows the BBox of the SPP-SegNet. The predicted output and the overlays are from the proposed SPP-SegNet. It can be noted that the proposed SPP-SegNet outperforms the standard SegNet, as the segmented result is much closer to the predicted output compared to the SegNet architecture. The confusion matrix of the proposed method is depicted in Figure 4.

**Figure 5:**
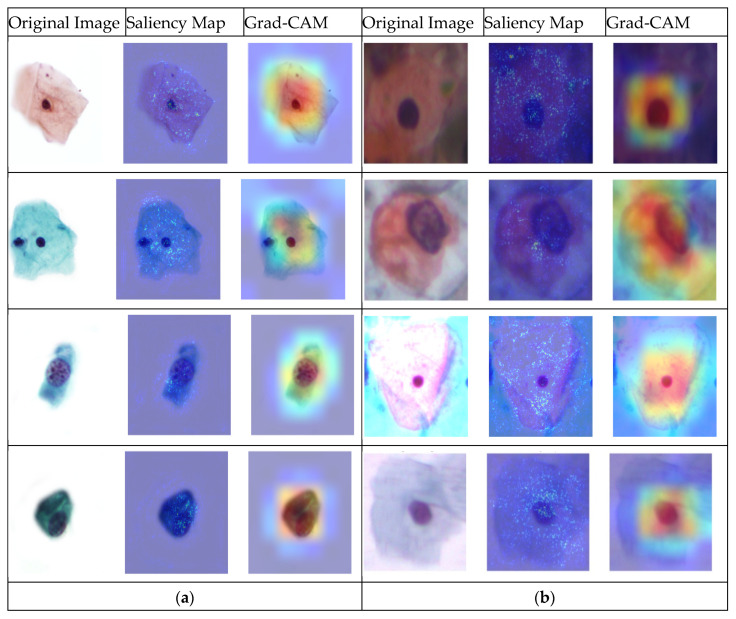
Saliency map and Grad-CAM visualization: (**a**) Pomeranian and (**b**) SIPaKMeD dataset.

As shown in Figure 4, six images were misclassified in the Pomeranian dataset, and fifteen images were misclassified in the SIPaKMeD binary classification. In this paper, we used both saliency maps and Grad-CAM to visualize and understand the regions of the image that influence the model’s decisions. A very popular technique for deriving per-decision explanations is through the generation of a saliency map, which visualizes the importance of different regions in the input image that contributed to the final decision. Saliency maps highlight the key image areas identified by the deep learning model [27]. Grad-CAM computes the weighted sum of feature maps based on their gradients, followed by the application of a ReLU activation function to generate the class activation map [7]. This map highlights the regions in the image that contributed most significantly to the model’s classification decision. By focusing on these high-impact areas, Grad-CAM provides valuable insights into the model’s attention and the reasoning behind its predictions. The visualization using saliency and Grad-CAM is depicted in Figure 5.

As shown in Figure 5, the first column displays the original cervical cell images, the second column shows the saliency maps, and the third column presents the Grad-CAM visualizations for both datasets—Pomeranian (left side) and SIPaKMeD (right side). The Pomeranian dataset includes NSIL samples in the first and second rows, and HSIL samples in the third and fourth rows. In the SIPaKMeD samples, the top two rows represent abnormal cells, while the bottom two show normal cells. The saliency maps and Grad-CAM visualizations consistently highlight regions with key morphological features. For NSIL samples (Pomeranian, rows 1 and 2), the model focuses primarily on the cytoplasmic region with less emphasis on the nucleus. In contrast, for HSIL samples (rows 3 and 4), there is strong model attention on the nuclear region, particularly areas showing nuclear enlargement and irregular contours, which are hallmarks of high-grade lesions. Similarly, in the SIPaKMeD dataset, the top two abnormal samples show heatmaps concentrated on the enlarged and hyperchromatic nuclei, consistent with pathological expectations. The normal cells in the bottom rows exhibit diffuse attention, indicating that the model is focusing more uniformly across the cytoplasm. These results demonstrate that the model is learning to prioritize diagnostically significant regions, particularly the nuclear morphology, when making predictions. This alignment with pathologist-defined criteria supports the model’s interpretability and reliability. It also validates that the visual explanation methods (saliency and Grad-CAM) reflect meaningful features, offering confidence in their use for model evaluation and refinement.

Table 5 presents a comparative analysis of our classification results with prior studies on the SIPaKMeD dataset. As shown in Table 5, our proposed SPP-SegNet model demonstrates superior performance in cervical cell segmentation compared to several existing methods. Traditional techniques such as FCM [3] achieved 85% precision on the Herlev dataset, while more advanced models like SE-ATT-Unet [16] and U-Net [17] reported precision scores of 92.32% and 95.04%, respectively. Although these models contribute valuable improvements, they often lack mechanisms for effective multiscale context aggregation. In contrast, our SPP-SegNet integrates spatial pyramid pooling and atrous convolution to enhance multiscale feature extraction, leading to higher accuracy (98.53% on Pomeranian and 94.15% on SIPaKMeD) and IoU scores (95.75% and 95.08%, respectively). These results confirm the model’s effectiveness and robustness in handling varying cervical cell morphologies and imaging challenges.

For classification, several deep learning architectures have been employed for the five-class task on the SIPaKMeD dataset. ResNet50 [28], a deep residual network designed to mitigate the vanishing gradient problem through skip connections, achieved 95% accuracy, demonstrating strong feature extraction capabilities. A basic CNN [29] reached 93% accuracy but lacked additional optimization techniques. To enhance feature selection, a CNN with principal component analysis (PCA) [30] was employed, improving accuracy slightly to 94% by reducing dimensionality and focusing on the most significant features. Another approach, CNN with a convolutional block attention module (CBAM) [31] incorporated attention mechanisms to refine spatial and channel feature selection, resulting in 92.8% accuracy with improved precision and recall. Additionally, for the two-class classification task, DenseNet121 [7], which leverages dense connectivity to strengthen gradient flow, achieved 95% in all performance metrics. In comparison, our proposed SE-DenseNet201 for two-class classification significantly outperforms previous methods, achieving 99% accuracy, precision, recall, and F1-score. Likewise, our approach for five-class classification attains 96% across all metrics, surpassing the existing methods and demonstrating the effectiveness of our model in capturing complex feature representations. Overall, the comparison demonstrates that our proposed models outperform existing methods, particularly in both binary and multi-class classification.

As a result, the proposed SPP-SegNet is effective at segmenting the entire cell from the background. Furthermore, the SE-DenseNet201 model achieves good results, particularly with the BBox method. This result may be helpful to cytotechnicians in identifying abnormal cells, allowing for a more accurate and efficient analysis. Improved segmentation can potentially aid in early detection and better diagnosis of cellular abnormalities.

## 5. Conclusions

In conclusion, the SPP-SegNet architecture significantly improves cervical cell segmentation in a single-cell Pap smear image, surpassing the performance of the standard SegNet model. By incorporating SPP and atrous convolution techniques, SPP-SegNet enhances the extraction of multiscale features, leading to more accurate and robust segmentation. Additionally, SE-DenseNet201 achieves better results in classifying cervical cells, with the segmentation output based on the BBox serving as input for classification. The results from both the Pomeranian and SIPaKMeD datasets demonstrate that the proposed method outperforms the standard method in all performance metrics. This improved segmentation accuracy is critical for the early detection of cervical cancer, as it helps to identify abnormal cells with greater precision. The proposed model shows great potential to automate Pap smear screening, reduce the workload of cytotechnicians, and minimize human error in diagnosing cervical abnormalities. Overall, SPP-SegNet and SE-DenseNet201 represent a valuable advance in the field of medical image analysis, offering a promising solution for the detection of cervical cancer. A limitation of the current study is the use of isolated single-cell images, which may not fully capture the complexity of real clinical samples. Future work will focus on extending the proposed method to multi-cell and whole-slide images, where challenges such as overlapping cells, heterogeneous staining, and large-scale image size must be addressed for effective clinical application. Additionally, clinical validation involving pathologists or cytotechnicians is needed to evaluate the system’s real-world performance and impact on diagnostic accuracy and workflow efficiency.

## Figures and Tables

**Table 1:** Details of the datasets used in this study.

Dataset	Category	No. of Img	Categories	Image Size	Total Image
Pomeranian	HSIL	124		1130 × 1130	419
	LSIL	61			
	NSIL	234			
SIPaKMeD	Dyskeratotic	813	Abnormal	different	4049
	Koilocytotic	825			
	Metaplastic	793			
	Parabasal	787	Normal		
	Superficial–Intermediate	831			

**Table 2:** Segmentation results using the Pomeranian and SIPaKMeD dataset.

Dataset	Method	Accuracy	Precision	Recall	IoU
Pomeranian	Standard SegNet	97.86%	91.9%	90.31%	94.55%
	SPP-SegNet	98.53%	96.41%	91.32%	95.75%
SIPakMeD	Standard SegNet	90.95%	92.40%	90.05%	92.89%
	SPP-SegNet	94.15%	93.87%	94.94%	95.08%
Herlev	Standard SegNet	86.74%	91.05%	90.35%	90.33%
	SPP-SegNet	87.58%	91.95%	91.67%	91.13%

**Table 3:** Classification results using the Pomeranian and SIPaKMeD dataset in the ablation study.

Dataset	Method	Resent50V2				Densenet121				Densenet201			
		Acc	Pre	Rec	F1	Acc	Pre	Rec	F1	Acc	Pre	Rec	F1
Pomeranian	Only classification	81%	77%	81%	79%	87%	87%	87%	85%	90%	90%	90%	90%
	ROI	83%	87%	83%	84%	85%	84%	85%	84%	86%	86%	86%	83%
	BBox	85%	85%	85%	85%	88%	88%	88%	88%	88%	88%	88%	88%
SIPaKMeD Multicell	Only classification	90%	91%	90%	90%	92%	91%	91%	91%	91%	91%	91%	91%
	ROI	87%	86%	87%	86%	90%	90%	90%	90%	90%	90%	90%	90%
	BBox	89%	89%	89%	89%	91%	91%	91%	91%	91%	91%	91%	91%
SIPaKMeD Binary	Only classification	96%	96%	96%	96%	97%	97%	97%	97%	98%	98%	98%	98%
	ROI	93%	93%	93%	93%	94%	94%	94%	94%	94%	94%	94%	94%
	BBox	94%	94%	94%	94%	96%	96%	96%	96%	96%	96%	96%	96%
Herlev Binary	Only classification	87%	88%	87%	87%	88%	88%	88%	88%	88%	88%	88%	88%
	ROI	84%	84%	84%	84%	85%	85%	85%	85%	86%	86%	86%	86%
	BBox	86%	86%	86%	86%	85%	85%	85%	85%	87%	87%	87%	87%

**Table 4:** Classification results using the Pomeranian and SIPaKMeD dataset.

Dataset	Methods	Accuracy	Precision	Recall	F1 Score
Pomeranian	SE-DesneNet201 ROI	87%	87%	87%	87%
	SE-DesneNet201 BBox	93%	93%	93%	93%
SIPaKMeD Multiclass	SE-DesneNet201 ROI	94%	94%	94%	94%
	SE-DesneNet201 BBox	96%	96%	96%	96%
SIPaKMeD Binary	SE-DesneNet201 ROI	97%	97%	97%	97%
	SE-DesneNet201 BBox	99%	99%	99%	99%
Herlev Binary	SE-DesneNet201 ROI	89%	90%	89%	88%
	SE-DesneNet201 BBox	90%	91%	90%	90%

**Table 5:** Comparison of our results from the previous study using the SIPaKMeD dataset.

Ref	Dataset	Task	Class	Methods	Accuracy	Precision	Recall	F1 Score	IoU
[3]	Herlev	Segmentation	-	FCM	-	85%	-	-	-
[16]	Private data	Segmentation	-	SE-ATT-Unet	-	92.32%	-	-	-
[17]	TCT image	Segmentation	-	U-Net	-	95.04%	96.19%	-	91.60%
[28]	SIPaKMeD	Classification	5	ResNet50	95%	-	-	-	-
[29]	SIPaKMeD	Classification	5	CNN	93%	-	-	-	-
[30]	SIPaKMeD	Classification	5	CNN + PCA	94%	-	-	-	-
[31]	SIPaKMeD	Classification	5	CNN_CBAM	92.8%	93%	92.8%	92.8%	-
[7]	SIPaKMeD	Classification	2	DenseNet121	95%	95%	95%	95%	-
Ours	SIPaKMeD	Classification	5	SE-DenseNet201	96%	96%	96%	96%	-
Ours	SIPaKMeD	Classification	2	SE-DenseNet201	99%	99%	99%	99%	-
Ours	Pomeranian	Segmentation	-	SPP-SegNet	98.53%	96.41%	91.32%	-	95.75%
Ours	SIPaKMeD	Segmentation	-	SPP-SegNet	94.15%	93.87%	94.94%	-	95.08%

## Data Availability

The SIPaKMeD dataset is publicly available.
